# BUILDING BRIDGES, CATALYZING RESEARCH, EMPOWERING ALL AGES: NIA SESSION FOR EARLY-CAREER RESEARCHERS

**DOI:** 10.1093/geroni/igad104.0971

**Published:** 2023-12-21

**Authors:** Amy Kelley, Amy Kelley

## Abstract

The National Institute on Aging (NIA) at the National Institutes of Health, Department of Health and Human Services, supports biomedical and behavioral research with a lifespan focus. NIA research seeks to understand the basic processes of aging, improve prevention and treatment of diseases in later life, and improve the health of older persons, in addition to an emphasis on Alzheimer’s disease and related dementias. NIA supports a variety of training and career development opportunities for students, junior faculty, early-career investigators, and emerging scholars. In this session, we will provide an overview of NIA-funded research, followed by a presentation on funding mechanisms and strategies to consider when applying for extramural grants, particularly for early-career scientists at multiple career stages. Finally, attendees will be able to join any number of breakout group discussions (led by extramural NIA training staff and program officials) to discuss programs, initiatives, mechanisms, and/or policies specific to various career paths.

